# Underreporting of Quality Measures and Associated Facility Characteristics and Racial Disparities in US Nursing Home Ratings

**DOI:** 10.1001/jamanetworkopen.2023.14822

**Published:** 2023-05-23

**Authors:** Prachi Sanghavi, Zihan Chen

**Affiliations:** 1 Biological Sciences Division, Department of Public Health Sciences, University of Chicago, Chicago, Illinois

## Abstract

**Importance:**

The US government rates nursing homes and reports the underlying quality measures on the Nursing Home Care Compare (NHCC) website. These measures are derived from facility-reported data, which research indicates to be substantially underreported.

**Objective:**

To assess the association between nursing home characteristics and reporting of major injury falls and pressure ulcers, which are 2 of 3 specific clinical outcomes reported by the NHCC website.

**Design, Setting, and Participants:**

This quality improvement study used hospitalization data for all Medicare fee-for-service beneficiaries between January 1, 2011, and December 31, 2017. Hospital admission claims for major injury falls and pressure ulcers were linked with facility-reported Minimum Data Set (MDS) assessments at the nursing home resident level. For each linked hospital claim, whether the nursing home had reported the event was determined and reporting rates were computed. The distribution of reporting across nursing homes and the associations between reporting and facility characteristics were examined. To assess whether nursing homes reported similarly on both measures, the association between reporting of major injury falls and pressure ulcers within a nursing home was estimated, and racial and ethnic disparities that might explain the observed associations were investigated. Small facilities and those that were not included in the sample continuously in each year of the study period were excluded. All analyses were performed throughout 2022.

**Main Outcomes and Measures:**

Two nursing home–level MDS reporting rates, stratified by long-stay vs short-stay population or by race and ethnicity, were used: fall reporting rate and pressure ulcer reporting rate.

**Results:**

The sample included 13 179 nursing homes where 131 000 residents (mean [SD] age, 81.9 [11.8] years; 93 010 females [71.0%]; 81.1% with White race and ethnicity) experienced major injury fall or pressure ulcer hospitalizations. There were 98 669 major injury fall hospitalizations, of which 60.0% were reported, and 39 894 stage 3 or 4 pressure ulcer hospitalizations, of which 67.7% were reported. Underreporting for both conditions was widespread, with 69.9% and 71.7% of nursing homes having reporting rates less than 80% for major injury fall and pressure ulcer hospitalizations, respectively. Lower reporting rates were associated with few facility characteristics other than racial and ethnic composition. Facilities with high vs low fall reporting rates had significantly more White residents (86.9% vs 73.3%), and facilities with high vs low pressure ulcer reporting rates had significantly fewer White residents (69.7% vs 74.9%). This pattern was retained within nursing homes, where the slope coefficient for the association between the 2 reporting rates was −0.42 (95% CI, –0.68 to –0.16). That is, nursing homes with more White residents had higher reporting rates for major injury falls and lower reporting rates for pressure ulcers.

**Conclusions and Relevance:**

Results of this study suggest widespread underreporting of major injury falls and pressure ulcers across US nursing homes, and underreporting was associated with the racial and ethnic composition of a facility. Alternative approaches to measuring quality need to be considered.

## Key Points

**Question:**

To what extent are nursing home characteristics associated with reporting rates of major injury falls and pressure ulcers on the Nursing Home Care Compare (NHCC) website?

**Findings:**

In this quality improvement study involving 13 179 nursing homes, underreporting of major injury falls and pressure ulcers to the NHCC website was widespread across nursing homes and was associated with few facility characteristics other than racial and ethnic composition. Nursing homes with more White residents had high reporting rates for major injury falls, whereas facilities with more Black residents had higher reporting rates for pressure ulcers.

**Meaning:**

The findings of this study suggest that given the widespread underreporting on the NHCC website of 2 patient safety indicators across nursing homes, alternative approaches to measuring nursing home quality need to be considered.

## Teaser

This quality improvement study assesses facility-reported rates of major injury falls and pressure ulcers in nursing homes across the US and investigates whether nursing home characteristics are associated with underreporting of these measures.

## Introduction

Improving nursing home care has been a national priority in the US for decades, with public reporting at the center of the federal government’s efforts.^1,2^ The Nursing Home Care Compare (NHCC) website rates homes on a 5-point scale based on state inspections, staffing information from payroll data, and quality-of-care measures largely as reported by facilities.^3^ Quality measures have long generated suspicion, and recent work by Sanghavi et al^4^ and Chen et al^5^ found substantial underreporting nationally on at least some quality indicators. This finding raises several questions: How widespread is underreporting? Are some facilities more likely to underreport? Do nursing homes similarly underreport across indicators? Accurate measurement is required for effective surveillance and evaluation; hence, improving understanding of the variations in facility-reported data is crucial.

Past work has found associations between nursing home–reported data, called the Minimum Data Set (MDS),^6^ and other quality-of-care measures from alternative data sources, such as consumer complaints, inspection violations, hospital readmissions, and rehabilitation outcomes, although these comparisons have been between aggregate measures.^7,8,9,10,11,12,13,14^ Government reports have compared MDS assessments with medical records and documented large inconsistencies.^15,16,17^ However, these reports have generally been based on small samples and relied heavily on MDS itself as a starting point, thereby missing clinical events that were altogether unreported. As partial acknowledgment of these discrepancies, the Centers for Medicare & Medicaid Services (CMS) Five-Star Quality Rating System added 5 claims-based measures after 2016 and assigns lower weights to the quality measures domain in the overall star ratings, even though these are the only indicators that directly attempt to measure specific patient outcomes.^3^

Findings from 2 large-scale, resident-level investigations have helped confirm that inaccuracies in facility-reported data are substantial for at least 2 quality measures: major injury falls and pressure ulcers.^4,5^ These studies used hospital admission claims of Medicare beneficiaries to identify nursing home residents with major injury falls or pressure ulcers and assessed whether the facility had appropriately reported the event to CMS. Their authors found that only 58% of major injury falls and 55% of pressure ulcers were reported.^4,5^ Additionally, in examining racial disparities in reporting at the resident and facility levels, the authors found that major injury falls among Black residents were reported to CMS at a much lower rate than major injury falls among White residents.^4,5^

Thus far, research on the accuracy of MDS has focused on average reporting across facilities and mostly considered measures separately.^15,16,17,18^ A 2003 study^18^ based on 219 nursing homes revealed considerable variation in reporting accuracy across facilities, although no specific facility characteristic was associated with reporting quality. In this study, we examined variations in reporting across nursing homes and facility factors. The 2 measures, major injury falls and pressure ulcers, may be related since mobility can increase the risk for major injury falls and decrease the risk for pressure ulcers.^19,20^ To assess whether nursing homes that reported poorly on 1 measure also reported poorly on the other measure (that is, whether facilities have a reporting behavior), we estimated the association between reporting of major injury falls and reporting of pressure ulcers within a nursing home. Since past work has described the associations between socially constructed racial and ethnic groups and these conditions^4,5,21,22,23,24,25,26,27^ but not that institutional racism in nursing homes^28^ may affect reporting practices, we also investigated the reporting rates along this dimension.

## Methods

### Data Sources

We used hospitalization records from the MedPAR (Medicare Provider Analysis and Review) file and MDS 3.0 assessments for 100% of Medicare fee-for-service beneficiaries between January 1, 2011, and December 31, 2017. We obtained demographic, enrollment, and chronic conditions data from the Medicare Master Beneficiary Summary Files. These files were the source of the race and ethnicity variable, which were initially self-reported by beneficiaries and then further processed by the Research Triangle Institute to improve identification of Asian and Hispanic individuals. The institutional review board of the Biological Sciences Division of the University of Chicago approved this quality improvement study and waived the informed consent requirement because research could not practicably be carried out without the waiver. We followed the Standards for Quality Improvement Reporting Excellence (SQUIRE) reporting guideline.^29^

Facility characteristics were obtained from the Certification and Survey Provider Enhanced Reports (CASPER) data set, which is based on inspections, and the publicly available LTCFocus^30^ data set for the study period. We identified facility rurality by zip code using information from the Federal Office of Rural Health Policy.^31^ Additionally, quarterly quality measures and star ratings (which ranged from 1 to 5, with 5 indicating the highest rating) on the NHCC website were obtained from CMS^32^ and the means across the years were calculated.

We excluded small facilities (bottom 10th percentile) and those that were not included in the sample continuously in each year of the study period (eAppendix 1 in Supplement 1). The final sample included 13 179 nursing homes.

### Linked Hospital Admissions and Nursing Home Assessments

We followed the approach of 2 prior studies that linked hospital admissions and MDS assessments to create resident-level reporting data sets on major injury falls and pressure ulcers.^4,5^ These studies contained a detailed explanation of the linkage process and published the code online. For major injury falls, we extended the code to include newer years of data. eAppendix 2 in Supplement 1 describes the code.

Nursing homes are required by CMS to complete discharge assessments, including the major injury falls and pressure ulcer items under study, when a resident has been admitted to a hospital. Thus, following the algorithms of past work, we linked MDS discharge assessments to Medicare inpatient hospital records for residents who were discharged from a nursing home to go to a hospital for treatment of a major injury fall or stage 3 or 4 pressure ulcer, which was determined from the primary diagnosis on the hospital claim (eAppendix 1 in Supplement 1). We allowed linkages within 1 day of the nursing home discharge and hospital admission dates. We further required residents to return to the same nursing home within 1 day of the hospital discharge to maximize the possibility that the nursing home was familiar with the individual’s hospitalization, even though this was not a reporting requirement. The final linked data sets included information from the claims and assessments as well as beneficiary characteristics, such as short-stay vs long-stay status (eAppendix 2 in Supplement 1). Specifically, residents were identified as having a short-stay status if they had a 5-day prospective payment system MDS assessment within 100 days prior to hospitalization; otherwise, the residents were considered to have a long-stay status.

### Variables

For each facility, we obtained from LTCFocus the zip code, number of beds, occupancy rate, registered nurse (RN) hours per resident day, and an indicator of whether the nursing home was hospital-based. Data on the number of deficiencies and an indicator of whether a facility had an on-site physician during inspection were extracted from CASPER.

We created other facility-level variables based on all residents who were Medicare beneficiaries by linking MDS to the full sample of Medicare beneficiaries and not just individuals who were hospitalized for major injury falls or pressure ulcers. With the full sample of nursing home residents for each facility and year within the study period (2011 to 2017), we computed the percentages of White residents, Black residents, residents with dual eligibility for Medicare and Medicaid, and residents with Alzheimer disease and related dementias. Sample sizes of other races and ethnicities in most nursing homes were small, making comparisons difficult; hence, we focused on White and Black residents. We also created indicators for long-stay and short-stay status for these residents based on whether the total number of days in the facility exceeded 100 days and separately analyzed these populations. The means of all variables across the years were calculated.

### Outcomes

Table 1 shows MDS items related to major injury falls and pressure ulcers in this analysis. Item J1900C on major injury falls is used by the NHCC website to create a quality measure of the percentage of long-stay residents experiencing 1 or more falls with major injury. Since the NHCC website does not report on a short-stay major injury falls quality measure, we excluded short-stay fall reporting rates from this study. Items M0300B1, M0300C1, and M0300D1 on pressure ulcers by severity are used by the NHCC website to create a quality measure of the percentage of high-risk long-stay residents with pressure ulcers and are used as a prerequisite for a separate quality measure of the percentage of short-stay residents with new or worsened pressure ulcers. These measures are also used by the Five-Star Quality Rating System.

**Table 1.** Minimum Data Set 3.0 Items on Major Injury Falls and Pressure Ulcers^a^

Item^b^	Description	Coding instruction	Possible responses
J1900C	Number of falls since admission, entry or reentry, or prior assessment	Major injury: bone fractures, joint dislocations, closed head injuries with altered consciousness, subdural hematoma.	0, 1, ≥2
M0300B1	Current number of unhealed pressure ulcers at stage 2	Partial thickness loss of dermis presenting as a shallow open ulcer with a red or pink wound bed without slough. May also present as an intact or open or ruptured blister.	Number of pressure ulcers at each stage
M0300C1	Current number of unhealed pressure ulcers at stage 3	Full thickness tissue loss. Subcutaneous fat may be visible, but bone, tendon, or muscle is not exposed. Slough may be present but does not obscure the depth of tissue loss. May include undermining and tunneling.	Number of pressure ulcers at each stage
M0300D1	Current number of unhealed pressure ulcers at stage 4	Full thickness tissue loss with exposed bone, tendon, or muscle. Slough or eschar may be present on some parts of the wound bed. Often includes undermining and tunneling.	Number of pressure ulcers at each stage

^a^ Data were from Centers for Medicare & Medicaid Services.^6^

^b^ These items are used by the Nursing Home Care Compare to create quality measures and assign 5-star ratings. Item J1900C is used to create a measure of percentage of long-stay residents experiencing 1 or more major injury falls. Items M0300B1, M0300C1, and M0300D1 are used to create a measure of percentage of high-risk long-stay residents with pressure ulcers and to construct the quality measure of the percentage of short-stay residents with new or worsened pressure ulcers.

We created 2 nursing home–level reporting rates that were stratified by long-stay vs short-stay population and, in some cases, also by race and ethnicity. The first was the fall reporting rate, defined as the percentage of major injury falls reported on the J1900C item of all the major injury fall hospitalizations that, according to the algorithm, required reporting based on MDS rules. The second was the pressure ulcer reporting rate, defined as the percentage of pressure ulcers reported on item M0300B1, M0300C1, or M0300D1 of all the stage 3 or 4 pressure ulcer^33^ hospitalizations that, according to the algorithm, required reporting.

### Statistical Analysis

First, we stratified the facilities by reporting rate using cutoffs of 50% and 80% for both major injury falls and pressure ulcers and then computed mean facility characteristics for each stratum. Second, we assessed the association between reporting of major injury falls and reporting of pressure ulcers among long-stay residents. For comparison, we assessed the association between reporting of pressure ulcers among long-stay vs short-stay residents. Thus, the first analysis compared different outcomes in the same population, and the second analysis compared the same outcome in different populations. Since sharing facility-level data in some cases would have violated the data use agreement, we conducted this analysis by calculating mean facility data at the state level. Based on prior evidence of racial disparities in reporting,^4,5^ we further analyzed how associations between reporting rates of major injury falls and pressure ulcers varied by the racial and ethnic composition within the facility.

To assess the associations between reporting rates and the underlying frequencies of fall and pressure ulcer events, we calculated hospitalization rates for each condition and facility. To continue the analysis of disparities by racial and ethnic groups, we studied long-stay hospitalization rates for major injury falls and pressure ulcers by facility racial and ethnic composition and within-facility racial and ethnic groups. We also stratified nursing homes by tertiles of the distributions of hospitalization rates for each condition, and we computed mean facility characteristics in each stratum.

All statistical tests were 2-sided and significance was set at an α = .05. All analyses were conducted throughout 2022 using Python, version 3.8 (Python Software Foundation), and R, version 4.1.1 (R Foundation for Statistical Computing).

## Results

Of the 13 179 nursing homes included in the study, 12 668 had at least 1 major injury fall hospitalization among long-stay residents, 9234 had at least 1 pressure ulcer hospitalization among long-stay residents, and 9954 had at least 1 pressure ulcer hospitalization among short-stay residents. We identified 98 669 major injury fall hospitalizations, of which 60.0% were reported, and 39 894 stage 3 or 4 pressure ulcer hospitalizations, of which 67.7% were reported.

Among the 131 000 long-stay residents who experienced major injury fall or pressure ulcer hospitalization, the mean (SD) age was 81.9 (11.8) years. There were 93 010 females (71.0%) and 37 990 males (29.0%), and 0.5% of the residents had American Indian or Alaska Native, 1.4% had Asian, 11.8% had Black, 4.9% had Hispanic, 81.1% had White, and 0.4% had other or unknown race and ethnicity.

Table 2 summarizes the facility characteristics stratified by reporting rates for fall and pressure ulcers (low, medium, and high) among long-stay residents. Underreporting for both conditions was widespread, with 69.9% and 71.7% of nursing homes having reporting rates less than 80.0% for major injury falls and pressure ulcer hospitalizations, respectively. For the fall reporting rate, each facility had at least 1 event; for the pressure ulcer reporting rate, each facility had at least 3 events. The low reporting rate was 33.0% and the high reporting rate was 94.3% for major injury falls. Pressure ulcer reporting was similar, with 34.2% as the low rate and 94.5% as the high rate.

**Table 2.** Characteristics of Nursing Homes Stratified by Reporting Rate Levels for Long-Stay Residents^a^

Nursing home variables^b^	Nursing home fall reporting rate					Nursing home pressure ulcer reporting rate				
	Low	Medium	High	Low-medium difference	Low-high difference	Low	Medium	High	Low-medium difference	Low-high difference
No. of nursing homes (%)	3928 (31.0)	4927 (38.9)	3813 (30.1)	NA	NA	1353 (28.0)	2113 (43.7)	1366 (28.3)	NA	NA
No. of events (%)	25 520 (25.9)	46 571 (47.2)	26 578 (26.9)	NA	NA	7039 (20.9)	15 984 (47.5)	10 656 (31.6)	NA	NA
Reporting and event rate										
Reporting rate, %^c^	33.0	68.4	94.3	NA	NA	34.2	69.2	94.5	NA	NA
No. of hospitalizations per 100 residents^d^	1.2	1.5	1.3	−0.4	−0.1	0.8	1.1	1.2	−0.3	−0.4
NHCC website measures^e^										
Overall rating	3.08	3.10	3.22	−0.02	−0.15	2.90	2.83	2.77	0.07	0.13
Quality rating	3.69	3.51	3.52	0.18	0.18	3.57	3.49	3.41	0.07	0.16
Survey rating	2.69	2.74	2.84	−0.05	−0.15	2.58	2.54	2.48	0.04	0.10
No. of deficiencies	6.4	5.9	5.8	0.5	0.7	6.4	6.6	6.7	−0.2	−0.3
Staffing rating	2.98	3.08	3.21	−0.09	−0.22	2.85	2.81	2.87	0.05	−0.02
Long-stay pressure ulcer quality measure rating	NA	NA	NA	NA	NA	6.25	7.56	8.23	−1.31	−1.98
Long-stay fall quality measure rating	2.63	3.51	3.71	−0.89	−1.08	NA	NA	NA	NA	NA
Nursing home characteristics										
No. of beds	119.6	123.9	106.8	−4.3	12.8	137.8	148.3	146.0	−10.5	−8.3
Occupancy rate, %	82.6	83.1	83.4	−0.5	−0.9	83.7	84.0	83.5	−0.3	0.3
Rural, No.(%)	907 (23.1)	1557 (31.6)	1533 (40.2)	NA	NA	313 (23.1)	444 (21.0)	298 (21.8)	NA	NA
Region										
Northeast, No. (%)	613 (26.4)	957 (41.3)	749 (32.3)	NA	NA	240 (22.6)	484 (45.7)	336 (31.7)	NA	NA
Midwest, No. (%)	1097 (27.4)	1481 (37.0)	1425 (35.6)	NA	NA	314 (29.7)	454 (43.0)	288 (27.3)	NA	NA
South, No. (%)	1533(32.9)	1995 (42.8)	1130 (24.3)	NA	NA	660 (30.2)	943 (43.1)	585 (26.7)	NA	NA
West, No. (%)	685 (40.6)	494 (29.3)	509 (30.2)	NA	NA	139(26.3)	232 (43.9)	157 (29.7)	NA	NA
Hospital-based facility, No. (%)	43 (1.1)	74 (1.5)	130 (3.4)	NA	NA	14(1.0)	21 (1.0)	29 (2.1)	NA	NA
Physician on site, No. (%)	3853 (98.1)	4848 (98.4)	3741 (98.1)	NA	NA	1337 (98.8)	2079 (98.4)	1346 (98.5)	NA	NA
RN h per 100 resident days	41.7	41.8	45.6	−0.1	−3.9	38.8	39.6	42.7	−0.8	−3.9
Percentage of Black residents^f^	16.3	10.0	8.0	6.3	8.4	16.5	19.8	20.2	−3.3	−3.8
Percentage of White residents^f^	73.3	83.9	86.9	−10.6	−13.6	74.9	70.8	69.7	4.1	5.2
Percentage of residents with dual eligibility for Medicare and Medicaid^f^	56.7	53.1	51.8	3.6	4.9	59.3	60.4	61.5	−1.1	−2.2
Percentage of residents with ADRD^g^	61.3	62.0	59.5	−0.7	1.7	63.5	63.1	62.3	0.3	1.2

Abbreviations: ADRD, Alzheimer disease and related dementias; NA, not applicable; NHCC, Nursing Home Care Compare; RN, registered nurse.

^a^ The cutoffs for low, medium, and high reporting rates were 50% and 80%. The fall sample by reporting rates included nursing homes with at least 1 fall hospitalization from 2011 to 2017. The pressure ulcer sample by reporting rates included nursing homes with at least 3 pressure ulcer hospitalizations from 2011 to 2017. eAppendix 1 and eTable 2 in Supplement 1 show results of a sensitivity analysis in which all nursing homes with at least 1 pressure ulcer hospitalization from 2011 to 2017 were included. Data were from MDS 3.0 assessment, Medicare hospital admission data, and the NHCC website.^3,6^

^b^ The nursing home variables were calculated for each nursing home year and then the means were calculated across 2011 to 2017, except for reporting rates and number of hospitalizations per 100 residents.

^c^ The reporting rates were calculated as the number of hospitalizations for which the primary diagnosis on the hospital claim was reported on Minimum Data Set (MDS) 3.0 assessment divided by the total number of hospitalizations among long-stay residents for each nursing home from 2011 to 2017.

^d^ The number of hospitalizations per 100 residents was calculated as the number of hospitalizations divided by the number of Medicare fee-for-service beneficiaries among long-stay residents and multiplied by 100 for each nursing home from 2011 to 2017.

^e^ Used the Five-Star Ratings, which ranged from 1 to 5, with 5 indicating the highest rating.

^f^ The percentage of White or Black residents or residents with dual eligibility for Medicare and Medicaid was calculated from a linkage between MDS 3.0 assessment and the Medicare Master Beneficiary Summary File. Other racial and ethnic groups in the sample were Asian, Black, Hispanic, North American Native, other, and unknown.

^g^ The percentage of residents with ADRD was calculated from a linkage between MDS 3.0 assessment and the Medicare Master Beneficiary Summary File: Chronic Conditions segment.

Nursing homes with a high fall reporting rate compared with nursing homes with low and medium fall reporting rates were more likely to be in rural areas (40.2% vs 23.1% and 31.6%) and hospital-based (3.4% vs 1.1% and 1.5%) and had more RN hours per 100 resident days (45.6 vs 41.7 and 41.8), higher percentages of White residents (86.9% vs 73.3% and 83.9%), and lower percentages of residents with dual eligibility for Medicare and Medicaid (51.8% vs 56.7% and 53.1%). For the NHCC website measures, moving from the low to the high fall reporting rate categories, the overall (3.08 to 3.22), survey (2.69 to 2.84), and staffing (2.98 to 3.21) ratings increased, whereas the quality rating (3.69 to 3.52) decreased. For pressure ulcers, differences across the categories of reporting rate levels were less pronounced, although the facilities in the high reporting rate level had more RN hours per 100 resident days compared with facilities in the low and medium reporting rate levels (42.7 vs 38.8 and 39.6), fewer White residents (69.7% vs 74.9% and 70.8%), and more residents with dual eligibility for Medicare and Medicaid (61.5% vs 59.4% and 60.4%). For the NHCC website measures, moving from the low to the high pressure ulcer reporting rate categories, the overall (2.90 to 2.77), survey (2.58 to 2.48), and quality (3.57 to 3.41) ratings all decreased, whereas the staffing rating did not exhibit a clear pattern.

The Figure presents the association between state-level pressure ulcer reporting rates in long-stay residents vs short-stay residents (panel A) and the association between reporting rates of major injury falls vs pressure ulcers among long-stay residents (panel B). Reporting rates of pressure ulcers among long-stay residents increased with higher reporting of pressure ulcers among short-stay residents, whereas reporting rates of pressure ulcers among long-stay residents decreased with higher reporting rates of major injury falls among long-stay residents. The slope coefficient from the ordinary least-squares estimate was 0.23 (95% CI, 0.03-0.43) for panel A and −0.42 (95% CI, –0.68 to –0.16) for panel B. In addition, states with a percentage of White residents that was higher than the national mean had lower reporting rates for pressure ulcers but higher reporting rates for major injury falls among long-stay residents. Clustering by race and ethnicity was not observable in the pressure ulcer reporting rates between short-stay and long-stay residents.

**Figure.**
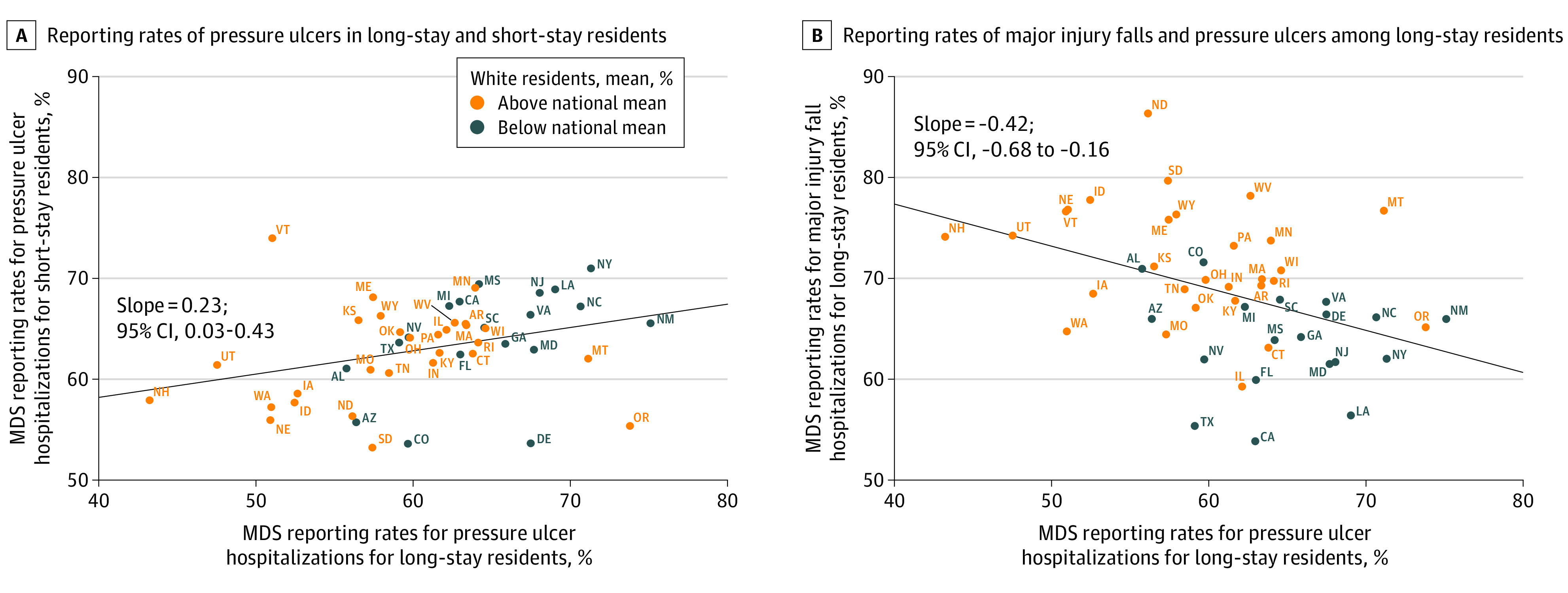
Nursing Home Reporting of Major Injury Falls and Pressure Ulcers on Minimum Data Set (MDS) Assessments by State From 2011 to 2017 Data were from MDS 3.0 assessment,^6^ Medicare hospital admission data, and Master Beneficiary Summary Files. Alaska, Hawaii, and the District of Columbia were excluded from these plots due to small sample sizes, which would have violated the data use agreement. The mean percentage of White residents at nursing homes was 83%. Other racial and ethnic groups in the sample were Asian, Black, Hispanic, North American Native, other, and unknown.

Table 3 shows the mean hospitalization rates per 100 long-stay Medicare beneficiaries for major injury falls and pressure ulcers for the Black, overall, and White populations, stratified by facility racial and ethnic composition. Generally, pressure ulcer rates and fall rates exhibited opposite patterns. Within the same level of facility racial and ethnic composition, the mean (SD) fall rates among White residents were between 2.2 and 2.5 times higher than the fall rates among Black residents (eg, 1.51 [0.74] vs 0.68 [4.53]), whereas pressure ulcer rates among Black residents were between 1.8 and 2.5 times higher than the pressure ulcer rates among White residents (eg, 0.73 [5.45] vs 0.29 [0.38]). As the facility-level percentage of White residents decreased, overall fall rates and fall rates among White and Black residents decreased, whereas the pressure ulcer rates increased across all groups. Findings were similar in a sensitivity analysis that excluded facilities with fewer than 50 Black or White residents (eAppendix 1 and eTable 1 in Supplement 1).

**Table 3.** Mean Number of Hospitalizations per 100 Long-Stay Resident Years for Major Injury Falls and Pressure Ulcers by Race and Race Composition From 2011 to 2017^a^

Nursing home level of White residents^b^	Mean (SD)					
	Major injury falls			Stage 3 or 4 pressure ulcers		
	Overall^c^	White residents^d^	Black residents^e^	Overall^c^	White residents^d^	Black residents^e^
High percentage	1.50 (0.74)	1.51 (0.74)	0.68 (4.53)	0.30 (0.38)	0.29 (0.38)	0.73 (5.45)
Medium percentage	1.37 (0.73)	1.44 (0.78)	0.58 (1.75)	0.44 (0.54)	0.40 (0.53)	0.88 (2.79)
Low percentage	1.03 (0.71)	1.29 (0.99)	0.54 (1.49)	0.86 (0.96)	0.66 (0.95)	1.21 (2.06)

^a^ Data were from Minimum Data Set 3.0 assessment, Medicare hospital admission data, and the Nursing Home Care Compare website.^3,6^

^b^ The cutoffs for high, medium, and low percentages of White residents were 95% and 79%, the 66th and 33rd percentiles, of the White residents vs residents with other racial and ethnic composition distribution. Other racial and ethnic groups in the sample were Asian, Black, Hispanic, North American Native, other, and unknown. Nursing homes with no White or Black residents were excluded from the sample, resulting in a sample with 11 134 facilities.

^c^ The overall number of hospitalizations per 100 long-stay resident years was calculated as the number of hospitalizations divided by the total Medicare fee-for-service beneficiaries for White or Black residents and multiplied by 100 for each nursing home from 2011 to 2017.

^d^ The number of hospitalizations per 100 long-stay resident years for White residents was calculated as the number of hospitalizations for White residents divided by the total Medicare fee-for-service White beneficiaries and multiplied by 100 for each nursing home from 2011 to 2017.

^e^ The number of hospitalizations per 100 long-stay resident years for Black residents was calculated as the number of hospitalizations for Black residents divided by the total Medicare fee-for-service Black beneficiaries and multiplied by 100 for each nursing home from 2011 to 2017.

When grouped by levels of hospitalization rates (hospitalizations per 100 Medicare beneficiaries), facilities with high fall rates, compared with low and medium rates, were more likely to be rural (38.2% vs 23.4% and 31.1%), were less likely to be hospital-based (1.5% vs 3.2% and 1.9%), had fewer RN hours per 100 resident days (40.5 vs 48.3 and 42.8), had higher percentages of White residents (87.8% vs 73.4% and 81.4%), and had lower percentages of residents with dual eligibility for Medicare and Medicaid (51.7% vs 55.9% and 54.0%) (Table 4). For the NHCC website measures, moving from facilities with low to high fall rates, the quality rating decreased (3.72 to 3.45), but the overall, survey, and staffing ratings did not follow a clear pattern. Facilities with high pressure ulcer rates, compared with low and medium rates, were less likely to be rural (21.9% vs 38.7% and 33.1%), were less likely to be hospital-based (1.4% vs 3.6% and 1.6%), had fewer RN hours per 100 residents days (41.3 vs 48.2 and 42.0), had lower percentages of White residents (70.7% vs 88.7% and 84.2%), and had higher percentages of residents with dual eligibility for Medicare and Medicaid (60.1% vs 48.0% and 53.1%). For the NHCC website measures, moving from facilities with low to high pressure ulcer rates, the overall (3.47 to 2.85), quality (3.69 to 3.48), survey (2.99 to 2.55), and staffing (3.38 to 2.85) ratings all decreased.

**Table 4.** Characteristics of Nursing Homes by Fall or Pressure Ulcer Hospitalization Rates for Long-Stay Residents^a^

Nursing home variables^c^	No. of hospitalizations per 100 Medicare beneficiaries^b^									
	Fall rate					Pressure ulcer rate				
	Low	Medium	High	Low-medium difference	Low-high difference	Low	Medium	High	Low-medium difference	Low-high difference
No. of nursing homes, (%)	4349 (33.0)	4085 (31.0)	4745 (36.0)	NA	NA	4343 (33.0)	4328 (32.8)	4508 (34.2)	NA	NA
No. of events, (%)	13 131 (13.3)	29 971 (30.4)	55 567 (56.3)	NA	NA	413 (1.0)	7996 (20.0)	31 485 (78.9)	NA	NA
Reporting and event rate										
Reporting rate, %^d^	62.2	65.1	67.9	−2.9	−5.7	57.4	58.6	66.8	−1.2	−9.4
No. of hospitalizations per 100 residents^e^	0.5	1.2	2.1	NA	NA	0.0	0.3	1.1	NA	NA
NHCC website measures^f^										
Overall rating	3.18	3.10	3.15	0.07	0.03	3.47	3.12	2.85	0.34	0.62
Quality rating	3.72	3.58	3.45	0.14	0.27	3.69	3.58	3.48	0.11	0.21
Survey rating	2.73	2.73	2.81	0.00	−0.08	2.99	2.75	2.55	0.24	0.44
No. of deficiencies	6.5	6.0	5.8	0.5	0.7	5.6	6.0	6.6	−0.4	−1.1
Staffing rating	3.12	3.06	3.12	0.06	0.00	3.38	3.08	2.85	0.30	0.53
Long-stay pressure ulcer quality measure	NA	NA	NA	NA	NA	4.82	5.78	7.56	−0.96	−2.74
Long-stay fall quality measure	2.44	3.15	4.08	−0.71	−1.64	NA	NA	NA	NA	NA
Nursing home characteristics										
No. of beds	115.5	122.1	111.9	−6.6	3.6	96.2	119.4	132.5	−23.15	−36.26
Occupancy rate, %	83.7	83.3	82.1	0.4	1.6	83.3	83.1	82.6	0.26	0.71
Rural, No. (%)	1018 (23.4)	1270 (31.1)	1813 (38.2)	NA	NA	1681 (38.7)	1433 (33.1)	987 (21.9)	NA	NA
Region										
Northeast No. (%)	807 (33.9)	812 (34.1)	765 (32.1)	NA	NA	704 (29.5)	781(32.8)	899 (37.7)	NA	NA
Midwest, No. (%)	1158 (28.0)	1257 (30.3)	1728(41.7)	NA	NA	1773 (42.8)	1295 (31.3)	1075 (25.9)	NA	NA
South, No. (%)	1314 (27.7)	1545 (32.6)	1885 (39.7)	NA	NA	1078 (22.7)	1722 (36.3)	1944 (41.0)	NA	NA
West, No. (%)	1070 (56.1)	471 (24.7)	367 (19.2)	NA	NA	788 (41.3)	530 (27.8)	590 (30.9)	NA	NA
Hospital-based facility, No. (%)	139 (3.2)	78 (1.9)	71 (1.5)	NA	NA	156 (3.6)	69 (1.6)	63 (1.4)	NA	NA
Physician on site, No. (%)	4279 (98.4)	4016 (98.3)	4655 (98.1)	NA	NA	4252 (97.9)	4259 (98.4)	4440 (98.5)	NA	NA
RN h per 100 resident days	48.3	42.8	40.5	5.5	7.8	48.2	42.0	41.3	6.23	6.90
Percentage of Black residents^g^	16.1	11.5	7.4	4.6	8.7	5.4	9.1	19.7	−3.64	−14.30
Percentage of White residents^g^	73.4	81.4	87.8	−8.0	−14.3	88.7	84.2	70.7	4.45	17.96
Percentage of residents with dual eligibility for Medicare and Medicaid^g^	55.9	54.0	51.7	1.9	4.2	48.0	53.1	60.1	−5.08	−12.16
Percentage of residents with ADRD^h^	57.5	61.2	62.8	−3.7	−5.2	58.3	61.5	61.9	−3.13	−3.54

Abbreviations: ADRD, Alzheimer disease and related dementias; NA, not applicable; NHCC, Nursing Home Care Compare; RN, registered nurse.

^a^ Data were from Minimum Data Set 3.0 assessment, Medicare hospital admission data, and the NHCC website.^3,6^

^b^ The cutoff for low, medium, and high fall hospitalization rates were 0.93 and 1.5 per 100 beneficiaries; the cutoff for pressure ulcer hospitalization rates were 0.14 and 0.51 per 100 beneficiaries. All nursing homes were included in the fall and pressure ulcer sample for hospitalization rates. eAppendix 1 and eTable 2 in Supplement 1 show results of a sensitivity analysis in which all nursing homes with at least 1 pressure ulcer hospitalization from 2011 to 2017 were included.

^c^ The nursing home variables were calculated for each nursing home year and then the means were calculated across 2011 to 2017, except for reporting rates and number of hospitalizations per 100 residents.

^d^ The reporting rates were calculated as the number of hospitalizations for which the primary diagnosis on the hospital claim was reported on Minimum Data Set 3.0 assessment divided by the total number of hospitalizations among long-stay residents for each nursing home from 2011 to 2017.

^e^ The number of hospitalizations per 100 residents were calculated as the number of hospitalizations divided by the number of Medicare fee-for-service beneficiaries among long-stay residents and multiplied by 100 for each nursing home from 2011 to 2017.

^f^ Used the Five-Star Ratings, which ranged from 1 to 5, with 5 indicating the highest rating.

^g^ The percentage of White or Black residents or residents with dual eligibility for Medicare and Medicaid was calculated from a linkage between Minimum Data Set 3.0 assessment and the Medicare Master Beneficiary Summary File. Other racial and ethnic groups in the sample were Asian, Black, Hispanic, North American Native, other, and unknown.

^h^ The percentage of residents with ADRD was calculated from a linkage between Minimum Data Set 3.0 assessment and the Medicare Master Beneficiary Summary File: Chronic Conditions segment.

## Discussion

A centerpiece of the US government’s efforts to improve nursing home care is the use of public reporting to inform consumers’ long-term care decisions and thereby increase competition among facilities. For 2 key quality measures that are facility-reported, major injury falls and pressure ulcers, we assessed the distribution of reporting rates across nursing homes, building on prior research that established the inaccuracy of national facility-reported data.^4,5^ The findings revealed that underreporting for both conditions was widespread, although at different levels within a nursing home. Differential reporting rates were associated with the racial and ethnic composition of a nursing home but few other facility characteristics. These findings have important implications beyond public reporting given that the same data are used for nursing home surveillance, priority setting, payment, and research.^6,34^

Although prior work has demonstrated poor reporting on major injury falls and pressure ulcers, it has not described the distribution of reporting across nursing homes.^4,5^ We found that only 60.0% of major injury falls were reported and 67.7% of stage 3 or 4 pressure ulcers were reported. As the Figure shows, states were spread across a wide range of reporting rates on both axes; in Table 2, as many nursing homes reported 33.0% major injury falls as reported 94.3% major injury falls, with a similar spread for pressure ulcers. These rates suggest that accurate reporting is achievable, although the circumstances that allow it remain unclear, and underreporting is common.

For major injury falls, lower rates of reporting were associated with lower overall, survey, and staffing ratings but generally higher quality ratings and a lower publicly reported fall rate. In the case of pressure ulcers, low reporting rates were associated with higher overall, quality, and survey ratings as well as a lower publicly reported pressure ulcer rate. Thus, low reporting rates were associated with a higher quality rating for both clinical events but exhibited opposite patterns for major injury falls vs pressure ulcers on other indicators.

Few facility characteristics were associated with higher reporting rates. For example, facilities with more RN hours per resident days had higher reporting rates. A higher staffing level was associated with higher quality of care,^35^ and more accurate reporting may be associated with better care. Associations were greater in magnitude between reporting rates and the percentage of White residents but were less pronounced between reporting rates and the percentage of residents with dual eligibility for Medicare and Medicaid. As the Figure demonstrates, the association between pressure ulcer reporting rates for short-stay vs long-stay residents had a positive direction and did not cluster by racial and ethnic composition (Figure). However, among long-stay residents, nursing homes with more White residents had higher reporting rates for major injury falls and lower reporting rates for pressure ulcers. It may be reasonable to expect differences in reporting of the same clinical event in 2 separate populations rather than reporting of different clinical events in the same population, but a change in the direction of association would not be expected. Therefore, we considered the role of race and ethnicity further by investigating the underlying event rates in each racial and ethnic group.

Hospitalization rates for major injury falls and pressure ulcers among long-stay residents were different by race (White vs Black residents) and by facility racial and ethnic composition. Within the strata of facility racial and ethnic composition, White residents had twice the fall rates of Black residents, whereas Black residents had twice the pressure ulcer rates of White residents. At the same time, as the percentage of White residents decreased in nursing homes, fall rates decreased and pressure ulcer rates increased in both racial and ethnic groups. Similar interfacility and intrafacility variations of pressure ulcer rates by race have been reported in other studies.^21,22,23,24,25,26,27,35,36,37^ Together, these findings indicate that these events occurred at different rates across racial and ethnic groups, which may be explained by the institutional racism that plagues long-term care facilities.^28^ For example, facilities with more White residents may minimize use of restraints, facilitate mobility, and provide regular repositioning, all of which could increase the risk of major injury falls while reducing the rate of pressure ulcers.^20,28,38^ Reflecting systemic racism in US society more broadly, disparities in chronic conditions that are associated with inequities in social, political, and economic access^28,39^ may also explain the differences in rates of major injury falls and pressure ulcers. For example, Black residents are more frequently diagnosed with obesity, a risk factor for pressure ulcers.^40,41,42^

Several findings help explain the negative direction of the association between the reporting rates of major injury falls and pressure ulcers within facilities. First, nursing homes in the US are segregated by race.^28,43,44,45,46^ Second, major injury falls occurred more frequently among White residents; severe pressure ulcers occurred more frequently among Black residents. Third, nursing homes more accurately reported events with higher hospitalization rates.

The associations between hospitalization rates and publicly reported ratings indicated that facilities with higher fall rates had lower quality ratings but otherwise did not have an association with other NHCC website ratings. However, facilities with high pressure ulcer reporting rates had lower ratings overall and in each domain.

### Limitations

This study has limitations. First, we relied on claims to estimate hospitalization rates, and although widely used, claims are generated for billing purposes. Second, we analyzed only events that led to hospitalization, although facilities were expected to report all events. Third, to meet the CMS cell suppression policy, we aggregated measures at the state level or in broad categories for sharing; however, most patterns also held at the facility level. Fourth, to avoid larger variances due to small denominators, we required nursing homes to have a minimum number of pressure ulcer hospitalizations to be included in the analysis of facility characteristics by reporting rates. In eAppendix 1 in Supplement 1, the results are shown without this restriction.

## Conclusions

The findings of this study have policy implications for the current efforts to improve nursing home quality. Underreporting of clinical events to CMS was widespread during the study period. Although CMS appears to recognize this situation, as evidenced by the lower weighting of quality measures in the ratings, the measures remain on public display. In other words, the quality measures are in limbo, but the public may be using these measures to make important life decisions. One approach to improving accuracy would be to use more objective data sources such as claims data, as CMS is increasingly doing. Even with alternative data sources, the study found that interpretation of quality measures needs to consider the racial and ethnic composition of nursing homes, which may affect both the prevalence of major injury falls and pressure ulcers and the accuracy of reporting.

## Notes

Supplement 1. **eAppendix 1.** Variations in Nursing Home-Reported Quality Data for U.S. Ratings
eReferences
**eTable 1.** Number of Hospitalizations per 100 Long-Stay Resident-Years for Major Injury Falls and Pressure Ulcers, by Race and Race Mix, 2011–2017 **eTable 2.** NHCC Measures and Nursing Home Characteristics by MDS Reporting Rates for Long-Stay Residents **eAppendix 2.** Code Description

Supplement 2.
Data Sharing Statement
