# Supplementary material for: Underreporting of Quality Measures and Associated Facility Characteristics and Racial Disparities in US Nursing Home Ratings
Source: JAMA Netw Open. 2023 May 23;6(5):e2314822. doi: 10.1001/jamanetworkopen.2023.14822 (PMC10208148; doi:10.1001/jamanetworkopen.2023.14822)
Supplement: Supplement 1. — eAppendix 1. Variations in Nursing Home-Reported Quality Data for U.S. Ratings eReferences eTable 1. Number of Hospitalizations per 100 Long-Stay Resident-Years for Major Injury Falls and Pressure Ulcers, by Race and Race Mix, 2011–2017 eTable 2. NHCC Measures and Nursing Home Characteristics by MDS Reporting Rates for Long-Stay Residents eAppendix 2. Code Description [file jamanetwopen-e2314822-s001.pdf]

Supplemental Online Content

Sanghavi P, Chen Z. Underreporting of quality measures and associated facility characteristics and racial disparities in US nursing home ratings. *JAMA Netw Open*. 2023;6(5):e2314822. doi:10.1001/jamanetworkopen.2023.14822

eAppendix 1. Variations in Nursing Home-Reported Quality Data for U.S. Ratings

eReferences

eTable 1. Number of Hospitalizations per 100 Long-Stay Resident-Years for Major Injury Falls and Pressure Ulcers, by Race and Race Mix, 2011–2017

eTable 2. NHCC Measures and Nursing Home Characteristics by MDS Reporting Rates for Long-Stay Residents

eAppendix 2. Code Description

This supplemental material has been provided by the authors to give readers additional information about their work.

© 2023 Sanghavi P et al. *JAMA Network Open.*

eAppendix 1. Variations in Nursing Home-Reported Quality Data for U.S. Ratings

A. Identification of major injury fall hospitalizations from MedPAR in 2016 and 2017

In the prior study on falls that we followed for linking assessments and hospitalizations, the study period was 2011 – 2015.[1] In this paper, we included data through 2017. We used the same methods as the published fall study, adapted from an algorithm developed by Kim et al.[2], to identify major injury fall hospitalizations from the Medicare Provider Analysis and Review (MedPAR) data, and cross walked fall-related ICD-9-CM codes to ICD-10-CM codes using CMS published mappings[3]. All diagnosis codes used can be found in the code link below.

B. Nursing home sample

Our initial sample consisted of 16,843 Medicare and/or Medicaid certified nursing homes from CASPER and LTCFocus during 2011 – 2017, and we imposed some restrictions for inclusion in the final sample. First, we excluded nursing homes with fewer than 4 observations (n=226) in CASPER or LTCFocus during the study period for more complete data of nursing home characteristics. Next, we excluded nursing homes that were not in the sample continuously from the beginning to the end of the study period (n=1,952). We also dropped nursing homes that failed to match with any nursing home from Minimum Data Set data (n=7). Since this study focused on hospitalizations for falls and pressure ulcers, which were rare events in nursing homes, we excluded small nursing homes with fewer than 37 residents, the 10th percentile of the sample distribution (n=1,469). Our final sample included 13,179 nursing homes.

C. Sensitivity analysis: Nursing home hospitalization rates for falls and pressure ulcers

Most nursing homes had predominantly White residents and thus had small numbers of residents who were Black. This could result in extreme estimates of hospitalization rates for falls and pressure ulcers among residents who were Black. Therefore, in this sensitivity analysis, we included nursing homes only if they had more than 50 residents who were White or Black to calculate the hospitalization rates. eTable 1 shows the results. Compared with Table 3 in the paper, the estimates changed a little, but the patterns remained the same: within the same level of nursing home race mix, fall hospitalization rates were higher among residents who were White than among residents who were Black, whereas pressure ulcer hospitalization rates were lower among residents who were White. In addition, nursing homes with lower percentages of White residents had lower fall rates but higher pressure ulcer rates.

D. Sensitivity analysis: Nursing home characteristics by pressure ulcer reporting rate

In our final sample, most nursing homes had small numbers of hospitalizations: among the nursing homes that had pressure ulcer hospitalizations, 47.7% (n=4402) experienced only one or two such events. Reporting rates based on such small denominators could have high uncertainties, as they can only assume the values of 0, 0.5, or 1 (both 0 and 0.5 would be categorized as low reporting rates according to our cutoff). Therefore, in the paper, we restricted the sample for nursing homes to those with at least three pressure ulcer hospitalizations during 2011 – 2017. In this sensitivity analysis, we present nursing home

© 2023 Sanghavi P et al. *JAMA Network Open*

characteristics by the level of pressure ulcer reporting accuracy based on all nursing homes with at least one such hospitalization. As expected, the estimates in the medium reporting group remain the same, whereas many of those in low and high reporting groups changed. Some patterns in eTable 2 become less clear in this sensitivity analysis. For example, in eTable 2, moving from the lowest reporting to the highest reporting for pressure ulcers, the overall rating decreased; however, in eTable 2, the overall rating fluctuated with no clear trend.

eReferences

1. Sanghavi P, Pan S, Caudry D. Assessment of nursing home reporting of major injury falls for quality measurement on nursing home compare. *Health Serv Res*. 2020;55(2):201-210. doi:10.1111/1475-6773.13247

2. Kim SB, Zingmond DS, Keeler EB, et al. Development of an algorithm to identify fall-related injuries and costs in Medicare data. *Inj Epidemiol*. 2016;3(1). doi:10.1186/s40621-015-0066-z

3. ICD-9-CM to and from ICD-10-CM and ICD-10-PCS Crosswalk or General Equivalence Mappings. NBER. https://www.nber.org/research/data/icd-9-cm-and-icd-10-cm-and-icd-10-pcs-crosswalk-or-general-equivalence-mappings. Accessed November 1, 2022.

© 2023 Sanghavi P et al. *JAMA Network Open*

eTable 1. Number of hospitalizations per 100 long-stay resident-years for major injury falls and pressure ulcers, by race and race mix, 2011 - 2017

**Nursing home percent of White residents [a]**	**Fall (mean, 25th percentile, 75th percentile)**			**Pressure ulcer (mean, 25th percentile, 75th percentile)**		
	**Overall [b]**	**Residents who were White [c]**	**Residents who were Black [d]**	**Overall [b]**	**Residents who were White [c]**	**Residents who were Black [d]**
High	1.40 (0.98, 1.77)	1.52 (1.04, 1.93)	0.62 (0.00, 1.15)	0.54 (0.18, 0.71)	0.46 (0.14, 0.62)	0.99 (0.00, 1.49)
Medium	0.87 (0.46, 1.21)	1.24 (0.55, 1.81)	0.54 (0.00, 0.84)	1.12 (0.43, 1.51)	0.82 (0.00, 1.15)	1.37 (0.40, 1.92)
Low	1.23 (0.78, 1.60)	1.49 (0.91, 1.98)	0.54 (0.00, 0.90)	0.77 (0.27, 1.04)	0.61 (0.13, 0.81)	1.21 (0.00, 1.74)

[a] Nursing homes with fewer than 50 residents who were White or Black were excluded from the sample, resulting in a sample with 4,467 facilities. The cutoffs for high, medium, and low percent of White residents were 78% and 59%, %, the 33rd and 66th percentiles of the White vs. residents who were not White race-mix distribution.
[b] The overall number of hospitalizations per 100 long-stay resident-years was calculated as the number of hospitalizations divided by the number of total Medicare Fee-for-service beneficiaries for residents who were White or Black and multiplied by 100 for each nursing home from 2011 - 2017.
[c] The number of hospitalizations per 100 long-stay resident-years for residents who were White was calculated as the number of hospitalizations for residents who were White divided by the total Medicare Fee-for-service beneficiaries who were White and multiplied by 100 for each nursing home from 2011 - 2017.
[d] The number of hospitalizations per 100 long-stay resident-years for residents who were Black was calculated as the number of hospitalizations for residents who were Black divided by the total Medicare Fee-for-service beneficiaries who were Black and multiplied by 100 for each nursing home from 2011 - 2017.

© 2023 Sanghavi P et al. *JAMA Network Open*

eTable 2. NHCC measures and nursing home characteristics by MDS reporting rates for long-stay residents[a]

Nursing home variables [b]	Pressure ulcer		
	Low	Medium	High
**N**	3584	2113	3537
**Reporting and event rates**			
Reporting rate [c]	23.8%	69.2%	97.9%
Number of hospitalizations per 100 beneficiaries [d]	0.5	1.1	0.7
**NHCC measures**			
Overall rating	3.09	2.83	3.00
Quality rating	3.61	3.49	3.48
Survey rating	2.73	2.54	2.66
No. of deficiencies	6.1	6.6	6.3
Staffing rating	3.01	2.81	3.04
Long stay pressure ulcer quality measure	5.75	7.56	6.89
**Nursing home characteristics**			
No. of beds	120.6	148.3	122.2
Occupancy rate	82.8%	84.0%	82.7%
Rural (%)	29.0%	21.0%	29.9%
Region:			
Northeast (100%)	33.8%	27.3%	38.8%
Midwest (100%)	42.1%	18.6%	39.3%
South (100%)	38.4%	24.8%	36.8%
West (100%)	39.8%	20.3%	39.8%
Hospital-based facility	1.2%	1.0%	2.2%
Physician on site	98.5%	98.4%	98.5%
RN hours per 100 resident days	41.7	39.6	43.1
Percentage of White residents [e]	81.2%	70.8%	78.7%
Percentage of residents dually eligible for Medicare and Medicaid [e]	54.5%	60.4%	55.9%
Percentage of residents with ADRD [f]	61.4%	63.1%	61.1%

MDS: Minimum Data Set; NHCC: Nursing Home Care Compare; RN: Registered nurse; ADRD: Alzheimer's Disease and related disorder or senile dementia

[a] The cutoff for low, medium, and high reporting rates was 50% and 80%. This sample included nursing home with at least one pressure ulcer hospitalization for long-stay residents during 2011 - 2017.

[b] The nursing home variables were calculated for each nursing home-year and then averaged across 2011 - 2017, except for reporting rate and number of hospitalizations per 100 residents.

[c] The reporting rates were calculated as the total number of hospitalizations where the primary diagnosis on hospital claims were reported on MDS assessments divided by the total number of hospitalizations among long-stay residents for each nursing home from 2011 - 2017.

[d] The number of hospitalizations per 100 residents were calculated as the total number of hospitalizations divided by the total number of Medicare Fee-for-service beneficiaries among long-stay residents and multiplied by 100 in each nursing home from 2011 - 2017.

[e] The percentage of White residents or residents who were dually eligible for Medicare and Medicaid was calculated from a linkage between MDS 3.0 and the Medicare Master Beneficiary Summary File.

[f] The percentage of residents with Alzheimer's Disease and related disorder or senile dementia was calculated from a linkage between MDS 3.0 and the Medicare Master Beneficiary Summary File: Chronic Conditions Segment.

© 2023 Sanghavi P et al. *JAMA Network Open*

eAppendix 2. Code Description

Link
https://github.com/sanghavi-lab/nhc_reportings

Software
We used Python 3.8 and R 4.2.1 for this analysis.

Steps
1. Setup sample data for fall hospitalizations in 2016 and 2017

We used the same code from two studies previously published on falls and pressure ulcers (the authors of those studies publicly shared the code) to build the sample data for fall hospitalizations in 2016 and 2017 (the published study only included 2011 - 2015). We identified major injury fall hospitalizations from MedPAR data, and linked the claims to MDS assessments for residents who were discharged from nursing homes, admitted to the hospital within one day, and returned to the same nursing home within one day after hospital discharge.

Scripts used in this step were listed under the "fall" folder.

2. Create nursing home variables using MDS assessments data and MBSF data

By linking MDS data and MBSF data, we created several nursing home-level variables that were used in the paper: the number of Medicare fee-for-service residents in nursing homes stratified by short stay and long stay as well as by White and Non-White, the percent of White residents, the percent of residents dually eligible for Medicare and Medicaid, the percent of residents with ADRD. We also aggregated ratings and quality measures from Nursing Home Care Compare (NHCC) data for nursing homes. Below are the scripts in this step.

Script name	Input	Output
0_create_nh_variables_mbsf.py	MDS and MBSF and MBSF Chronic Conditions from 2011 - 2017	nh_medicare_res_count_shortlongstay.csv<br>nh_medicare_res_count_shortlongstay_race.csv<br>nh_variables_capser.csv
0_medicare_count_adrd.py	MDS and MBSF and MBSF Chronic Conditions from 2011 - 2017	nh_adrd.csv
0_create_nhc_data.py	Ratings and quality measure data from NHCC website	nh_nhc_measures.csv

3. Merge all MDS reporting data

This step combined MDS reporting data previously created for falls and pressure ulcers. Please see detailed descriptions and code in corresponding links. The script for this step is 1_merge_all_mds_item.py, and the output from this script was all_mds_items_data.csv.

4. Create nursing home sample data

This step created nursing home sample from CASPER and LTCfocus, linked MDS reporting data to nursing home characteristics, and transformed all nursing home-year variables to nursing home variables. Below are the scripts in this step.

Script name	Input	Output
2_create_nh_sample_casper_ltcfocus.R	CASPER and LTCfocus data from 2011 - 2017	casper_and_ltc_clean2.csv
3_create_final_nh_sample.py	all_mds_items_data.csv<br>casper_and_ltc_clean2.csv<br>nh_nhc_measures.csv<br>nh_medicare_res_count_shortlongstay.csv<br>nh_medicare_res_count_shortlongstay_race.csv<br>nh_variables_capser.csv<br>forhp-eligible-zips.csv	nh_characteristics_20221101.csv

5. Create exhibits

This script 4_create_exhibits.R used the final nursing home sample data from last step and created all exhibits published in the paper. Input files used were nh_characteristics_20221101.csv and all_mds_items_data.csv; output files were Exhibit 2 – 4 in the paper.

© 2023 Sanghavi P et al. *JAMA Network Open*
