# Supplementary material for: Underreporting of Quality Measures and Associated Facility Characteristics and Racial Disparities in US Nursing Home Ratings
Source: JAMA Netw Open. 2023 May 23;6(5):e2314822. doi: 10.1001/jamanetworkopen.2023.14822 (PMC10208148; doi:10.1001/jamanetworkopen.2023.14822)
Supplement: Supplement 2. — Data Sharing Statement [file jamanetwopen-e2314822-s002.pdf]

Data Sharing Statement

Sanghavi. Underreporting of Quality Measures and Associated Facility Characteristics and Racial Disparities in US Nursing Home Ratings. *JAMA Netw Open*. Published May 23, 2023. doi:10.1001/jamanetworkopen.2023.14822

Data
Data available: No

Additional Information
Explanation for why data not available: We have signed a Data Use Agreement with the Centers for Medicare and Medicaid Services that does not allow sharing of individual patient data.
